# Editorial: Recent advances in tree genetics and genomics: where we stand and where to go?

**DOI:** 10.3389/fpls.2023.1338728

**Published:** 2023-11-30

**Authors:** Malay Das, Jianjun Chen

**Affiliations:** ^1^Department of Life Sciences, Presidency University, Kolkata, India; ^2^Mid-Florida Research and Education Center, Environmental Horticulture Department, University of Florida, Apopka, FL, United States

**Keywords:** genetics and genomics, tree, molecular markers, genetic diversity, micropropagation, QTL

Trees contribute significantly to our economy. Unlike reference plants, such as *Arabidopsis* or rice that have been in the spotlight for the past few decades, trees and tree genomes have received limited attention. Studying the biology of trees poses many challenges including long generation time (perennialism), low and asynchronous seed germination rate, difficulty for genetic transformation, and absence of genomic data and mutants. Despite so many challenges, genomes of several tree species have been sequenced, comparative genomics will help us to understand the complexity of tree genetics and genomes.

The objective of this Research Topic was to update current research advances in tree genetics and genomics. A total of six articles were accepted into this Research Topic, which include *Camellia sinensis* (tea), *Casuarina equisetifolia* (Australian pine), *Cunninghamia lanceolata* (lamb.) hook (Chinese fir), *Phyllostachs edulis* (Moso bamboo), and *Torreya grandis* (Chinese Nutmeg Tree).

OVATE family proteins (OFPs) are a novel class of plant-specific transcription regulators modulating plant growth and development. Through genome-wide analysis of the OVATE gene family in tea (*Camellia sinensis*) plants, An et al. identified 26 members from cultivar tea and 13 from ancient tea plants. Many OVATE family members were highly expressed in young leaves and shoots, but their expression levels decreased with the maturation of leaves. The authors also found a key gene associated with the development of leaf area. These findings may suggest that many OFPs could be implicated in the regulation of leaf development of woody plants.

Australian pine (*Casuarina equisetifolia*) is a monoecious tree that is cultivated in tropical and subtropical regions for various purposes. An effective micropropagation method was developed by Ahmad et al. where nodal segments were cultured on Murashige and Skoog medium supplemented with BA and NAA. A maximum of 32 adventitious shoots were produced per explant. Scanning electron microscopy showed that shoots was directly regenerated from explants. RAPD and ISSR analyses showed that the regenerated plants were genetically uniform. This developed protocol could be used for large-scale multiplication and germplasm preservation of *C. equisetifolia*, thus meeting the growing demands of *C. equisetifolia* in the forest industry.

Population genetic structure and diversity are critical for marker-assisted breeding of tree species. Using RAD-Seq technology, Jing et al. developed 343,644 high-quality single nucleotide polymorphism (SNP) markers and investigated genetic structure and diversity of 233 Chinese fir (*Cunninghamia lanceolata*) individuals derived from the fourth cycle of selection. Result showed that the genetic diversity in the fourth cycle was high with the nucleotide diversity (Pi) of 0.003, and Ho and He of 0.215 and 0.233, respectively, suggesting that the genetic base had increased in breeding population. This work provided a foundation for further marker-assisted breeding of Chinese fir and other coniferous trees.

Scientifically speaking, bamboo is a member of the grass family Poaceae. However, from the forest view, bamboo is woody grass or bamboo tree. Moso bamboo (*Phyllostachys edulis*) is a versatile plant species that is widely used as a construction material in many countries. Del Giudice et al. re-sequenced eight *Phyllostachys* species and 18 natural accessions of *P*. *edulis* and generated a large set of functionally annotated molecular markers (SNPs and InDels). Subsequent genome-wide association analysis identified several candidate genes that were correlated with a mechanical property, which is of highly interest to structural engineers: its tensile strength normal to its fibers (i.e., splitting). The splitting strength is strongly related to the structural performance of bamboo, and it was associated with genomic regions that included several important candidate genes. These genes could be used for population genetic studies and fine genetic association analyses. This study provides the genetic architecture of a complex structural property trait, the splitting strength, in bamboo.

*Torreya grandis* is a dioecious species with high economical and ornamental values. Chen et al. conducted a QTL mapping of *T. grandis* using an open-pollination (OP) mapping strategy. Nearly 100 unrelated trees randomly chosen from the species’ natural distribution and their half-sib progeny were simultaneously genotyped. The authors reconstructed a high-density linkage map of 4,203 SNPs covering a total distance of 8,393.95 cM. They identified 13 QTLs for stem basal diameter growth and four QTLs for stem height growth in juvenile seedlings. The authors believed that those QTLs and their harboring genomic regions were evolutionarily relatively young, and the plants should better be utilized by clonal propagation rather than through seed propagation. Genetic results from the OP sampling strategy could provide guidance for genetic studies of other dioecious species.

How tree populations in the wild respond to anthropogenic disturbance has received increasing attention. Using *Ailanthus altissima*, Saina et al. investigated the effects of anthropogenic disturbance on genetic variability in natural and disturbed forests. The genetic diversity and population structure of *A. altissima* was analyzed by nuclear and chloroplast microsatellite markers. The genetic diversity across the 34 study populations based on EST-SSRs was moderate to high (HE= 0.547-0.772) with a mean HE of 0.680 (Saina et al.). Overall, genetic variation did not differ substantially across disturbed and undisturbed sites, there were small trends toward higher genetic diversity and population bottlenecks in disturbed forests. The authors concluded that disrupted ecosystems might display surprising genetic patterns that could be difficult to predict and should not be overlooked.

## Final remarks

Papers published in this Research Topic highlight some recent progress on tree genetics and genomics. Considering the majority of trees are economically important; more research is needed to explore their values through genetic and genomic analyses. Advancement in long read sequencing technology has enabled researchers to sequence large polyploid genomes and negate over-reliance on a limited, reference tree genome, such as Poplar or Eucalyptus. Yet, accurate analysis of large heterozygous tree genomes needs robust bioinformatics pipeline. It will further assist in understanding genotype and population-level genetic diversity, which is required to connect sequence diversity to phenotypic variations. Other areas where significant progress may be noticed in the next few decades include genome assisted selection and breeding to improve important woody traits, biodiversity conservation and effective forest management practices to tackle climate change.

## Author contributions

MD: Writing – review & editing. JC: Writing – original draft.

